# Menopausal Hormone Replacement Therapy and the Risk of Ovarian Cancer: A Meta-Analysis

**DOI:** 10.3389/fendo.2019.00801

**Published:** 2019-12-03

**Authors:** Yang Liu, Lan Ma, Xiaoling Yang, Jia Bie, Dongya Li, Chunyi Sun, Jie Zhang, Yushi Meng, Jie Lin

**Affiliations:** ^1^Department of Reproductive, The Second Affiliated Hospital of Kunming Medical University, Kunming, China; ^2^Department of Gynecology, The Second Affiliated Hospital of Kunming Medical University, Kunming, China; ^3^Department of Surgery, The Second Affiliated Hospital of Kunming Medical University, Kunming, China; ^4^Department of Oncology, The Second Affiliated Hospital of Kunming Medical University, Kunming, China

**Keywords:** ovarian cancer, menopause, hormone replacement therapy, meta-analysis, association

## Abstract

**Background:** Findings by epidemiologic studies on menopausal hormone replacement therapy (HRT) and the risk of ovarian cancer are inconsistent. This study aimed to assess the association of menopausal HRT with the risk of ovarian cancer by histological subtype.

**Methods:** A literature search was performed in PubMed, Web of Science, and EmBase for relevant articles published from inception to August 2018. Pooled relative risk ratios (RRs) with 95% confidence intervals (CIs) were determined with a random-effects model.

**Results:** Thirty-six studies involving 4, 229, 061 participants were included in this meta-analysis. The pooled RR of ovarian cancer was 1.29 (95%CI 1.19–1.40, *I*^2^ = 57.4%) for menopausal HRT. In subgroup analysis by study design, pooled RRs of ovarian cancer in cohort and case-control studies were 1.35 (95%CI 1.19–1.53) and 1.24 (95%CI 1.11–1.38), respectively. In subgroup analysis by continent, association of menopausal HRT with ovarian cancer was significant for North America (1.41 [1.23–1.61]), Europe (1.22 [1.12–1.34]), and Asia (1.76 [1.09–2.85]), but not Australia (0.96 [0.57–1.61]). Association differed across histological subtypes. Increased risk was only found for two common types, including serous (1.50 [1.35–1.68]) and endometrioid (1.48 [1.13–1.94]) tumors.

**Conclusion:** This meta-analysis suggests that menopausal HRT may increase the risk of ovarian cancer, especially for serous and endometrioid tumors.

## Introduction

Ovarian cancer is known as the most lethal genital system malignancy (1). It is also the fifth leading cause of cancer-related deaths in American women (1). In 2018, the estimated new ovarian cancer cases and deaths will be 22,240 and 14,070 in the US, respectively (1). In 2018, the age standardized incidence rate of ovarian cancer is 6.6 per 100,000 in world^1^. Ovarian cancer can be divided into five histologic subtypes: serous tumor, mucinous tumor, endometrioid tumor, clear cell tumor, and other type of ovarian cancer. And the different histologic types of ovarian cancer may has different protective factors or pathogenic factors. Breastfeeding (2) and oral contraceptives (3) have been confirmed as protective factors in ovarian cancer. However, other exposures such as obesity (4, 5), diabetes (6), miscarriage (7) and a family history of breast/ovarian cancer (8) are demonstrated risk factors for ovarian cancer.

Menopausal hormone replacement therapy (HRT) is widely used to improve postmenopausal symptoms and ward off bone loss. However, in the past few years, many epidemiological studies have revealed that HRT is associated with an increased risk of breast cancer (9, 10). Data regarding HRT and the risk of ovarian cancer are contradictory. According to several studies, HRT is associated with an increased risk of ovarian cancer (11–20). However, several studies found no relationship between them (21–37) and others found the positive association in individual histological subtype (38–46). Although the increased risk of ovarian cancer associated with menopausal HRT has been described previously in several meta-analyses, the histological subtype of ovarian cancer was not taken into account (47, 48). Until now, whether the effect of HRT on the risk of ovarian cancer differs by histological subtype is not completely known. Therefore, we performed the current meta-analysis to evaluate the effect of menopausal HRT on ovarian cancer risk by histological subtype.

## Materials and Methods

### Literature Search Strategy

We performed a literature search to identify relevant available articles from PubMed, Web of Science and EmBase from inception to August 2018 with no restrictions. Search terms included “hormone replacement therapy” (or “HRT”) and “ovarian cancer” (or “ovarian neoplasms” or “ovarian carcinoma” or “ovary cancer”). The reference lists of the included studies were also reviewed for potential relevant studies.

### Inclusion Criteria

Inclusion criteria were: (1) original report from observational studies; (2) menopausal HRT as the exposure of interest; (3) ovarian cancer as the outcome of interest; (4) relative risk ratio (RR) with 95% confidence interval (CI) provided. The most recent and complete study was selected if studies from the same population were repeated.

Two investigators searched and reviewed all relevant studies independently. Any disagreement was resolved by consensus with the involvement of a third reviewer.

### Data Extraction

The following information were extracted from each study by two investigators independently: first author's name, published year, country, study design, follow-up duration, age range or mean age at baseline, sample size and number of cases, histological subtype of ovarian cancer, the types of hormones used in the study population, RR (we presented all results as RR for simplicity) with 95%CI and adjustment for potential confounders. We extracted RRs adjusted for the most confounding factors in the original studies. We prioritized the RRs for highest vs. lowest duration category of HRT use. If the study did not provide RRs for highest vs. lowest duration category of HRT use, we extracted the RRs for “use vs. non-use.”

### Statistical Analysis

The Newcastle–Ottawa Scale was used to assess the quality of studies included in this meta-analysis. Pooled data were obtained as the inverse variance-weighted means of the logarithm of RRs with 95%CI to assess the associations of menopausal HRT and the risk of different histological subtypes of ovarian cancer, respectively. The DerSimonian and Laird random effects model (REM) was used to combine study-specific RRs (95%CIs). The *I*^2^ statistic was adopted to assess heterogeneity among studies (*I*^2^-values of 0, 25, 50, and 75% represented no, low, moderate and high heterogeneity, respectively). Meta-regression with restricted maximum likelihood estimation was performed to explore the important covariates that might have significant impact on between-study heterogeneity. Subgroup analyses were stratified on study design, geographic location and the types of hormones used in the study population. Sensitivity analysis was performed with one study removed at a time to assess whether the results could have been affected markedly by a single study. The funnel plot and Egger's test were performed to explore the small-study effect.

All statistical analyses were performed with STATA version 14.0 (Stata Corporation, College Station, TX, United States). All reported probabilities (*P*-values) were two-sided, with a statistical significance level of 0.05.

## Results

### Literature Search Results

**Figure 1:**
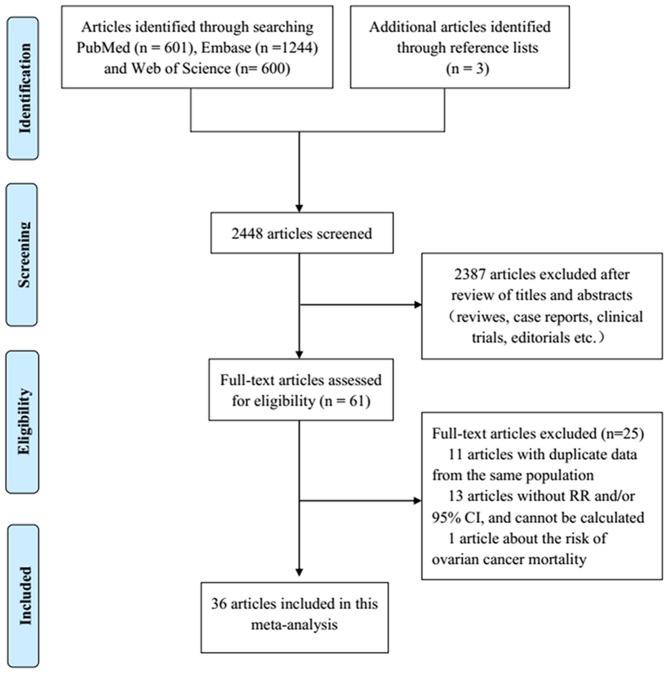
Flowchart of the study selection process based on PRISMA in this meta-analysis.

We identified 2,445 articles by literature search, of which 2,387 were excluded after title and abstract review (Figure 1). Three additional articles were found by searching the reference lists of included articles. Eleven articles with duplicate data from the same population, 13 reports without RR and/or 95%CI and one article assessing the risk of ovarian cancer mortality were excluded. Finally, 36 published articles were eligible for this meta-analysis.

### Characteristics of Studies

For the association of menopausal HRT with the risk of ovarian cancer, 34 articles (11–28, 30–42, 44–46) (15 cohort and 19 case-control studies) were included, involving 3,305,108 participants. The Newcastle-Ottawa Scale indicated that most of the studies included in this meta-analysis were of high quality (thirty of them scored more than seven). Among these studies, 15 were performed in Europe, 15 in North America, 2 in Asia and 2 in Australia. For the association of menopausal HRT and the risk of ovarian cancer by histological subtype, 12 studies (21, 29, 35, 38–46) assessing 1,193,201 participants were included for serous tumors, 10 reports (21, 38–46) evaluating 1,173,009 participants were included for endometrioid tumors, 9 studies (35, 38–45) assessing 1,089,421 participants were included for mucinous tumors, 5 reports (21, 39, 41, 43, 45) with 1,081,067 participants were included for clear cell tumors and 5 studies (38, 39, 41, 44, 45) evaluating 175,429 participants were included for other types of ovarian cancer. The detailed characteristics of the included studies are shown in Table 1.

**Table 1:** The detailed characteristics of the included studies.

**References**	**Country (year)**	**Age**	**Study design**	**Years of follow-up**	**Participants (cases)**	**Cancer type**	**Hormone type**	**RR (95% CI)**	**Adjustment for covariant**
Danforth et al. (46)	America (2007)	61.2 (mean)	Cohort	26 years	82,950 (389)	Ovarian cancer	HRT	1.41 (1.07, 1.86)	Age, parity, duration of oral contraceptive use, tubal ligation, age at natural menopause, age at menarche
Danforth et al. (46)	America (2007)	61.2 (mean)	Cohort	26 years	82,950 (233)	Serous tumors	HRT	1.66 (1.17, 2.36)	Age, parity, duration of oral contraceptive use, tubal ligation, age at natural menopause, age at menarche
Danforth et al. (46)	America (2007)	61.2 (mean)	Cohort	26 years	82,950 (60)	Endometrioid tumors	HRT	1.86 (0.89, 3.91)	Age, parity, duration of oral contraceptive use, tubal ligation, age at natural menopause, age at menarche
Bethea et al. (30)	America (2017)	37.8 ± 10.3	Cohort	18 years	59,000 (115)	Ovarian cancer	HRT	1.42 (0.75, 2.70)	Age, questionnaire cycle, parity, lactation, age at first birth, age at last birth, hysterectomy, tubal ligation, oral contraceptive use, educational HRT attainment, and BMI
Li et al. (25)	10 European countries (2015)	52.4 (median)	Cohort	11.7 years	367,903 (791)	Ovarian cancer	HRT	1.09 (0.92, 1.30)	Menopausal status, age at menopause, age at menarche, number of full-term pregnancies (FTPs), age at first FTP, duration of breast-feeding, number of miscarriages, unilateral ovariectomy, hysterectomy, HRT, OC use, IUD use, BMI, smoking status, alcohol consumption, and pre-existing diabetes
Soegaard et al. (38)	Denmark (2007)	35-79	Case control	NA	1,614 (50)	Mucinous tumors	HRT	0.71 (0.37, 1.36)	Age, pregnancy, additional pregnancies and duration of oral contraceptive use
Soegaard et al. (38)	Denmark (2007)	35-79	Case control	NA	1,907 (343)	Serous tumors	HRT	1.30 (1.00, 1.68)	Age, pregnancy, additional pregnancies and duration of oral contraceptive use
Soegaard et al. (38)	Denmark (2007)	35-79	Case control	NA	1,639 (75)	Endometrioid tumors	HRT	1.75 (1.07, 2.84)	Age, pregnancy, additional pregnancies and duration of oral contraceptive use
Soegaard et al. (38)	Denmark (2007)	35-79	Case control	NA	1,650 (86)	Other types of ovarian cancer	HRT	1.43 (0.90, 2.28)	Age, pregnancy, additional pregnancies and duration of oral contraceptive use
Soegaard et al. (38)	Denmark (2007)	35-79	Case control	NA	2,118 (554)	Ovarian cancer	HRT	1.30 (1.05, 1.61)	Age, pregnancy, additional pregnancies and duration of oral contraceptive use
Koskela-Niska et al. (39)	Finland (2013)	> 50	Case control	NA	15,283 (3,958)	Ovarian cancer	HRT	1.15 (0.99, 1.32)	Age and place of residence
Koskela-Niska et al. (39)	Finland (2013)	> 50	Case control	NA	7,333 (1,903)	Serous tumors	HRT	1.45 (1.20, 1.75)	Age and place of residence
Koskela-Niska et al. (39)	Finland (2013)	> 50	Case control	NA	2,901 (748)	Endometrioid tumors	HRT	1.25 (0.88, 1.76)	Age and place of residence
Koskela-Niska et al. (39)	Finland (2013)	> 50	Case control	NA	1,611 (417)	Mucinous tumors	HRT	0.35 (0.19, 0.67)	Age and place of residence
Koskela-Niska et al. (39)	Finland (2013)	> 50	Case control	NA	596 (155)	Clear cell tumors	HRT	0.72 (0.23, 2.29)	Age and place of residence
Koskela-Niska et al. (39)	Finland (2013)	> 50	Case control	NA	2,842 (735)	Other types of ovarian cancer	HRT	0.82 (0.56, 1.20)	Age and place of residence
Folsom et al. (11)	America (2004)	55-69	Cohort	15 years	31,381 (174)	Ovarian cancer	ERT	2.53 (1.44, 4.45)	Age, family history of ovarian cancer in a first- or second-degree relative, hysterectomy, unilateral oophorectomy, number of live births, physical activity index, pack-years of smoking, waist/hip ratio, and BMI
Risch (40)	Canada (1996)	Case: 59.5, control: 57.5 (mean)	Case control	NA	776 (212)	Serous tumors	ERT	2.03 (1.04, 3.97)	Age, number of full-term pregnancies, total years of oral-contraceptive use, and average lactation/pregnancy as continuous terms, and history of tubal ligation, hysterectomy, and mother/sister with breast cancer as dichotomous terms
Risch (40)	Canada (1996)	Case: 59.5, control: 57.5 (mean)	Case control	NA	637 (73)	Endometrioid tumors	ERT	2.81 (1.15, 6.89)	Age, number of full-term pregnancies, total years of oral-contraceptive use, and average lactation/pregnancy as continuous terms, and history of tubal ligation, hysterectomy, and mother/sister with breast cancer as dichotomous terms
Risch (40)	Canada (1996)	Case: 59.5, control: 57.5 (mean)	Case control	NA	604 (40)	Mucinous tumors	ERT	0.58 (0.08, 4.21)	Age, number of full-term pregnancies, total years of oral-contraceptive use, and average lactation/pregnancy as continuous terms, and history of tubal ligation, hysterectomy, and mother/sister with breast cancer as dichotomous terms
Risch (40)	Canada (1996)	Case: 59.5, control: 57.5 (mean)	Case control	NA	891 (327)	Ovarian cancer	ERT	1.77 (0.98, 3.20)	Age, number of full-term pregnancies, total years of oral-contraceptive use, and average lactation/pregnancy as continuous terms, and history of tubal ligation, hysterectomy, and mother/sister with breast cancer as dichotomous terms
Perri et al. (12)	Israeli (2015)	Case: 53.6 ± 10.3, control: 49.1 ± 13.4	Cohort	18 years	1,073 (175)	Ovarian cancer	HRT	1.98 (1.21, 3.25)	Mutation type, age at menarche, oral contraceptive use, parity, age at first pregnancy
Bakken et al. (26)	Norway (2004)	53.0 (mean)	Cohort	7 years	30,115 (74)	Ovarian cancer	HRT	1.30 (0.80, 2.00)	Age, BMI, smoking, ever use of OCs, time since menopause, parity and age at last birth
Mills et al. (41)	America (2005)	NA	Case control	NA	1,378 (256)	Ovarian cancer	HRT	1.39 (1.01, 1.93)	Age, race/ethnicity, duration of oral contraceptive use and breastfeeding
Mills et al. (41)	America (2005)	NA	Case control	NA	1,214 (92)	Serous tumors	HRT	1.61 (0.99, 2.60)	Age, race/ethnicity, duration of oral contraceptive use and breastfeeding
Mills et al. (41)	America (2005)	NA	Case control	NA	1,157 (35)	Endometrioid tumors	HRT	0.96 (0.44, 2.10)	Age, race/ethnicity, duration of oral contraceptive use and breastfeeding
Mills et al. (41)	America (2005)	NA	Case control	NA	1,138 (16)	Mucinous tumors	HRT	1.32 (0.40, 4.40)	Age, race/ethnicity, duration of oral contraceptive use and breastfeeding
Mills et al. (41)	America (2005)	NA	Case control	NA	1,134 (12)	Clear cell tumors	HRT	1.14 (0.27, 4.84)	Age, race/ethnicity, duration of oral contraceptive use and breastfeeding
Mills et al. (41)	America (2005)	NA	Case control	NA	1,149 (27)	Other types of ovarian cancer	HRT	1.30 (0.57, 2.97)	Age, race/ethnicity, duration of oral contraceptive use and breastfeeding
Purdie et al. (23)	Australia (1999)	18-79	Case control	NA	1,648 (793)	Ovarian cancer	HRT	1.20 (0.90, 1.60)	Age, education, area of residence, BMI, hysterectomy, tubal sterilization, talc use in perineal region, smoking status, duration of OCP use, parity and a family history of breast or ovarian cancer
Riman et al. (42)	Sweden (2002)	Case: 62.4 ± 7.4, control: 63.4 ± 7.1	Case control	NA	4,432 (642)	Ovarian cancer	ERT	2.10 (0.99, 4.48)	Age, parity,BMI (kg/m^2^), age at menopause, hysterectomy,duration of oral contraceptive use, and ever use of estrogen only (estrogen replacement therapy [ERT]) and continuous estrogen–progestin combinations (HRTcp) as categorized variables
Riman et al. (42)	Sweden (2002)	Case: 62.6 ± 7.3, control: 63.4 ± 7.1	Case control	NA	4,123 (333)	Serous tumors	ERT	2.51 (1.00, 6.34)	Age, parity,BMI (kg/m^2^), age at menopause, hysterectomy,duration of oral contraceptive use, and ever use of estrogen only (estrogen replacement therapy [ERT]) and continuous estrogen–progestin combinations (HRTcp) as categorized variables
Riman et al. (42)	Sweden (2002)	Case: 61.6 ± 7.6, control: 63.4 ± 7.1	Case control	NA	3,967 (177)	Endometrioid tumors	ERT	2.24 (0.64, 7.89)	Age, parity,BMI (kg/m^2^), age at menopause, hysterectomy,duration of oral contraceptive use, and ever use of estrogen only (estrogen replacement therapy [ERT]) and continuous estrogen–progestin combinations (HRTcp) as categorized variables
Riman et al. (42)	Sweden (2002)	Case: 62.5 ± 7.8, control: 63.4 ± 7.1	Case control	NA	3,850 (60)	Mucinous tumors	ERT	1.59 (0.19, 13.33)	Age, parity,BMI (kg/m^2^), age at menopause, hysterectomy,duration of oral contraceptive use, and ever use of estrogen only (estrogen replacement therapy [ERT]) and continuous estrogen–progestin combinations (HRTcp) as categorized variables
Kotsopoulos et al. (22)	America (2006)	Case: 62.7, control: 61.2 (mean)	Case control	NA	537 (162)	Ovarian cancer	HRT	0.93 (0.56, 1.56)	Parity, OC use and country of residence
Hempling et al. (21)	America (1997)	Case: 54.9, control: 54.9 (mean)	Case control	NA	1,255 (499)	Ovarian cancer	HRT	0.60 (0.30, 1.40)	Age at diagnosis, parity, oral contraceptive use, smoking history, family history of epithelial ovarian cancer, age at menarche, menopausal status, income, location, and education
Hempling et al. (21)	America (1997)	Case: 54.9, control: 54.9 (mean)	Case control	NA	NA	Serous tumors	HRT	1.20 (0.80, 1.70)	NA
Hempling et al. (21)	America (1997)	Case: 54.9, control: 54.9 (mean)	Case control	NA	NA	Clear cell tumors	HRT	1.10 (0.40, 3.40)	NA
Hempling et al. (21)	America (1997)	Case: 54.9, control: 54.9 (mean)	Case control	NA	NA	Endometrioid tumors	HRT	0.40 (0.20, 1.20)	NA
Sit et al. (24)	America (2002)	Case: 56.6, control: 55.7 (mean)	Case control	NA	1,410 (848)	Ovarian cancer	HRT	1.03 (0.69, 1.53)	Numbers of live births, family history of ovarian carcinoma,OC use, history of tubal ligation, and age at diagnosis
Mørch et al. (43)	Denmark (2012)	≥50	Cohort	8 years	909,946 (1,336)	Serous tumors	HRT	1.64 (1.41, 1.89)	Age, time period, number of births, educational level, and history of hysterectomy, sterilization, unilateral oophorectomy or salpingo-oophorectomy, endometriosis, and infertility
Mørch et al. (43)	Denmark (2012)	≥50	Cohort	8 years	909,946 (377)	Endometrioid tumors	HRT	1.81 (1.39, 2.36)	Age, time period, number of births, educational level, and history of hysterectomy, sterilization, unilateral oophorectomy or salpingo-oophorectomy, endometriosis, and infertility
Mørch et al. (43)	Denmark (2012)	≥50	Cohort	8 years	909,946 (293)	Mucinous tumors	HRT	0.74 (0.51, 1.08)	Age, time period, number of births, educational level, and history of hysterectomy, sterilization, unilateral oophorectomy or salpingo-oophorectomy, endometriosis, and infertility
Mørch et al. (43)	Denmark (2012)	≥50	Cohort	8 years	909,946 (159)	Clear cell tumors	HRT	0.81 (0.50, 1.32)	Age, time period, number of births, educational level, and history of hysterectomy, sterilization, unilateral oophorectomy or salpingo-oophorectomy, endometriosis, and infertility
Morch et al. (13)	Denmark (2009)	≥50	Cohort	8 years	909,946 (2,297)	Ovarian cancer	HRT	1.57 (1.26, 1.95)	Age, period of use, number of births, hysterectomy, sterilization, unilateral oophorectomy or salpingo-oophorectomy, endometriosis, infertility, and educational status
Wernli et al. (27)	America (2008)	40-79	Case control	NA	6,559 (751)	Ovarian cancer	HRT	1.24 (0.97, 1.60)	BMI, oral contraceptive use, tubal ligation, parity, family history of ovarian cancer, hysterectomy, and menopausal status
Urban et al. (14)	America (2015)	50-79	Cohort	12.3 years	74,786 (461)	Ovarian cancer	HRT	1.50 (1.23, 1.83)	Age and race
Tavani et al. (15)	Italy (2000)	Case: 54.0, control: 52.0 (mean)	Case control	NA	232 (93)	Ovarian cancer	HRT	1.80 (1.30, 2.60)	Age and area of residence
Lacey et al. (16)	America (2002)	56.6 (mean)	Cohort	13.4 years	44,241 (275)	Ovarian cancer	ERT	3.20 (1.70, 5.70)	Age, menopause type, and duration of oral contraceptive use
Simin et al. (17)	Sweden (2017)	≥40	Cohort	7 years	290,186 (573)	Ovarian cancer	HRT	1.09 (1.00, 1.19)	NA
Yang et al. (45)	America (2012)	Case: 62.8 ± 5.3, control: 61.8 ± 5.4	Cohort	Case: 5.1 years; control: 9.8 years	168,323 (849)	Ovarian cancer	HRT	1.57 (1.31, 1.89)	Age, oral contraceptive use, parity, menopausal hormone therapy
Yang et al. (45)	America (2012)	Case: 62.6 ± 5.4, control: 61.8 ± 5.4	Cohort	Case: 5.1 years; control: 9.8 years	168,323 (449)	Serous tumors	HRT	1.64 (1.27, 2.13)	Age, oral contraceptive use, parity, menopausal hormone therapy
Yang et al. (45)	America (2012)	Case: 61.0 ± 6.2, control: 61.8 ± 5.4	Cohort	Case: 5.1 years; control: 9.8 years	168,323 (78)	Endometrioid tumors	HRT	2.27 (1.26, 4.09)	Age, oral contraceptive use, parity, menopausal hormone therapy
Yang et al. (45)	America (2012)	Case: 63.5 ± 5.5, control: 61.8 ± 5.4	Cohort	Case: 5.1 years; control: 9.8 years	169,391 (37)	Mucinous tumors	HRT	0.50 (0.17, 1.42)	Age, oral contraceptive use, parity, menopausal hormone therapy
Yang et al. (45)	America (2012)	Case: 59.7 ± 6.2, control: 61.8 ± 5.4	Cohort	Case: 5.1 years; control: 9.8 years	168,323 (26)	Clear cell tumors	HRT	1.82 (0.64, 5.17)	Age, oral contraceptive use, parity, menopausal hormone therapy
Yang et al. (45)	America (2012)	Case: 63.9 ± 4.8, control: 61.8 ± 5.4	Cohort	Case: 5.1 years; control: 9.8 years	168,323 (255)	Other types of ovarian cancer	HRT	1.53 (1.11, 2.13)	Age, oral contraceptive use, parity, menopausal hormone therapy
Rossing et al. (18)	America (2007)	Case: 47.0, control: 48.0 (median)	Case control	NA	1,818 (715)	Ovarian cancer	ERT	1.60 (1.10, 2.50)	Age, county of residence, year of diagnosis/reference date, number of full-term pregnancies, and duration of hormonal contraception
Moorman et al. (44)	America (2005)	20-74	Case control	NA	734 (364)	Ovarian cancer	HRT	1.20 (0.80, 1.60)	Age, race, parity, tubal ligation, hysterectomy, BMI 1 year before interview, 1st degree family history of breast or ovarian cancer, breastfeeding, oral contraceptive use, and educational level
Moorman et al. (44)	America (2005)	20-74	Case control	NA	572 (216)	Serous tumors	HRT	2.00 (1.30, 3.10)	Age and race
Moorman et al. (44)	America (2005)	20-74	Case control	NA	421 (65)	Endometrioid tumors	HRT	1.00 (0.50, 2.00)	Age and race
Moorman et al. (44)	America (2005)	20-74	Case control	NA	382 (25)	Mucinous tumors	HRT	0.90 (0.30, 2.50)	Age and race
Moorman et al. (44)	America (2005)	20-74	Case control	NA	397 (40)	Other types of ovarian cancer	HRT	1.10 (0.50, 2.70)	Age and race
Beral et al. (19)	United Kingdom (2007)	57.2 ± 4.6	Cohort	5.3 years	948,576 (2,273)	Ovarian cancer	HRT	1.31 (1.12, 1.53)	Region of residence, socioeconomic group,time since menopause, parity, BMI, alcohol consumption, and use of oral contraceptives
Koskela-Niska et al. (39)	Finland (2013)	≥50	Cohort	12 years	224,015 (602)	Ovarian cancer	HRT	1.13 (0.74, 1.64)	Age
Rasmussen et al. (29)	Denmark (2017)	NA	Case control	NA	14,007 (885)	Ovarian cancer	HRT	1.34 (0.86, 2.09)	Age, tubal ligation, salpingectomy, hysterectomy, endometriosis, pelvic inflammatory disease, infertility, parity, and hormone replacement therapy
Chiaffarino et al. (31)	Italy (2001)	Case: 56.0, control: 57.0 (median)	Case control	NA	3,442 (1031)	Ovarian cancer	HRT	1.40 (0.80, 2.50)	Age, center education, parity, OC use. and family history of ovarian and breast cancer in first degree relative
Braem et al. (32)	Netherlands (2010)	Case: 62.0, control: 61.5 (mean)	cohort	16 years	2,706 (375)	Ovarian cancer	HRT	0.97 (0.69, 1.37)	Age, parity, duration of OC and HRT use
Pasalich et al. (33)	China (2013)	Case: 59.0 ± 5.6, control: 59.7 ± 6.4	Case control	NA	1,000 (500)	Ovarian cancer	HRT	1.05 (0.35, 3.21)	Age, smoking status, alcohol drinking, education, BMI,mutually adjusted for parity, oral contraceptive use, hormone replacement therapy, menopausal status, hysterectomy and family history of ovarian and/or breast cancer
Salazar-Martinez et al. (34)	Mexico (1999)	Case: 52.8, control: 54.6 (mean)	Case control	NA	752 (84)	Ovarian cancer	HRT	1.00 (0.36, 2.70)	Age, anovulatory index, smoking, diabetes mellitus, hypertension, physical activity, menopausal status, and body build index
Jordan et al. (35)	Australia (2007)	18-79	Case control	NA	885 (133)	Mucinous tumors	HRT	0.67 (0.28, 1.64)	Age, state of residence, education, parity, hysterectomy, smoking status
Jordan et al. (35)	Australia (2007)	18-79	Case control	NA	982 (230)	Serous tumors	HRT	0.71 (0.40, 1.27)	Age, state of residence, education, parity, hysterectomy, smoking status
Jordan et al. (35)	Australia (2007)	18-79	Case control	NA	1,115 (363)	Ovarian cancer	HRT	0.70 (0.42, 1.17)	Age, state of residence, education, parity, hysterectomy, smoking status
Polychronopoulou et al. (20)	Greece (1993)	<75	Case control	NA	389 (189)	Ovarian cancer	HRT	5.73 (1.07, 30.80)	Age, years of schooling, weight before the disease, age at menarche, parity and age at first birth
Adami et al. (36)	Sweden (1989)	54.5 (mean)	cohort	6.7 years	23,244 (64)	Ovarian cancer	HRT	0.96 (0.74, 1.23)	NA
Schneider et al. (37)	United Kingdom (2009)	51.3 ± 6.1	Case control	NA	602 (86)	Ovarian cancer	HRT	0.97 (0.61, 1.54)	Smoking status, BMI, use of oral contraceptives, progesterone preparations and vaginal estrogens

*RR, relative risk; CI, confidence interval; BMI, body mass index; NA, not available; HRT, hormone replacement therapy; ERT, estrogen replacement therapy*.

### Quantitative Synthesis

The association of menopausal HRT with the risk of ovarian cancer is summarized in Table 2.

**Table 2:** Summary risk estimates of the association between hormone replacement therapy and ovarian cancer.

**Subgroup**	**No. of studies**	**Pooled RR (95% CI)**	***I*^**2**^ (%)**	***P*_**heterogeneity**_**
All studies	34	1.29 (1.19–1.40)	57.4	<0.001
After excluding two studies (RR > 3.0)	32	1.27 (1.17–1.37)	52.1	<0.001
**Study design**				
Cohort studies	15	1.35 (1.19–1.53)	72.9	<0.001
Case control studies	19	1.24 (1.11–1.38)	30.4	0.103
**Hormones types**				
HRT	28	1.24 (1.15–1.35)	51.6	0.001
ERT	6	1.85 (1.28–2.66)	75.1	0.001
**Geographic location**				
North America	15	1.41 (1.23–1.61)	45.5	0.028
Europe	15	1.22 (1.12–1.34)	52.6	0.009
Asia	2	1.76 (1.09–2.85)	4.8	0.305
Australia	2	0.96 (0.57–1.61)	69.1	0.072

*RR, relative risk; CI, confidence interval; HRT, hormone replacement therapy; ERT, estrogen replacement therapy*.

**Figure 2:**
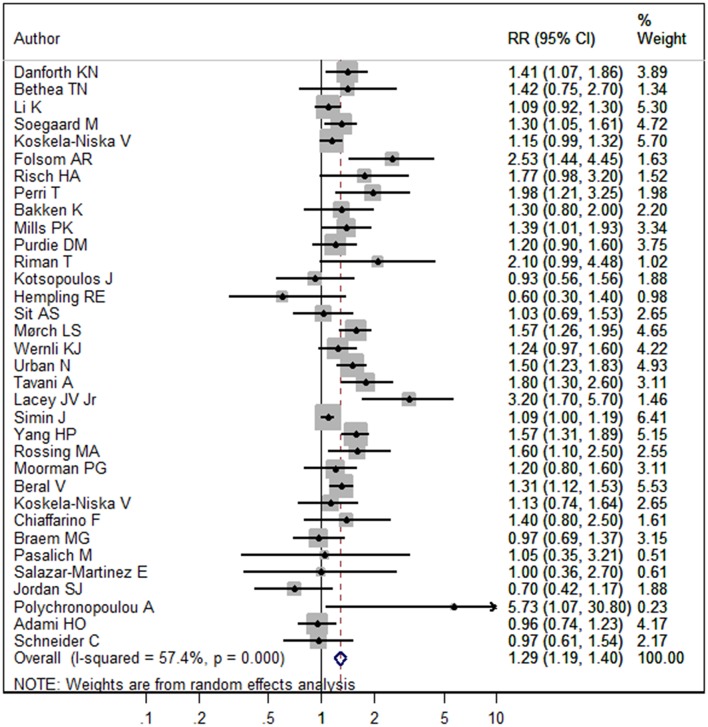
Forest plot of menopausal HRT and the risk of ovarian cancer. The size of a gray box is proportional to the weight assigned to the respective study, and horizontal lines represent 95% confidence intervals (CIs).

**Figure 3:**
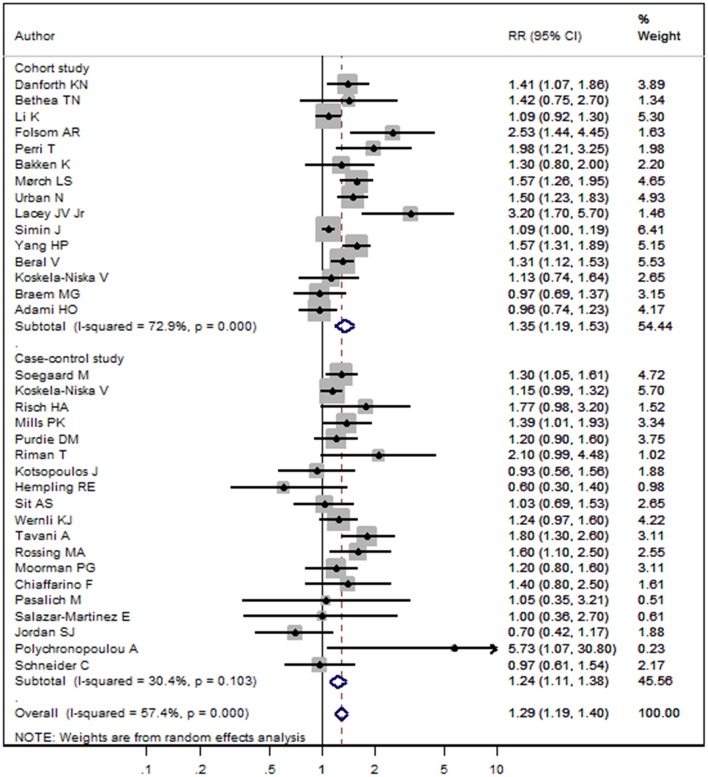
Forest plot of menopausal HRT and the risk of ovarian cancer in subgroup analysis stratified by study design. The size of a gray box is proportional to the weight assigned to the respective study, and horizontal lines represent 95% confidence intervals (CIs).

The pooled RR of menopausal HRT and the risk of ovarian cancer was 1.29 (95%CI 1.19–1.40, *I*^2^ = 57.4%, *P*_heterogeneity_ < 0.001, Figure 2). In subgroup analysis stratified by study design, pooled RRs in cohort and case-control studies were 1.35 (95%CI 1.19–1.53, *I*^2^ = 72.9%, *P*_heterogeneity_ < 0.001) and 1.24 (95%CI 1.11–1.38, *I*^2^ = 30.4%, *P*_heterogeneity_ = 0.103), respectively (Figure 3). In subgroup analysis stratified by geographic location, significant positive associations were found for North America (RR = 1.41, 95%CI 1.23–1.61, *I*^2^ = 45.5%, *P*_heterogeneity_ = 0.028), Europe (RR = 1.22, 95%CI 1.12–1.34, *I*^2^ = 52.6%, *P*_heterogeneity_ = 0.009), and Asia (RR = 1.76, 95%CI 1.09–2.85, *I*^2^ = 4.8%, *P*_heterogeneity_ = 0.305), but not Australia (RR = 0.96, 95%CI 0.57–1.61, *I*^2^ = 69.1%, *P*_heterogeneity_ = 0.072) (Figure S1). In subgroup analysis stratified by the hormones types, pooled RRs for HRT and ERT (estrogen replacement therapy) were 1.24 (95%CI 1.15–1.35, *I*^2^ = 51.6%, *P*_heterogeneity_ = 0.001) and 1.85 (95%CI 1.28–2.66, *I*^2^ = 75.1%, *P*_heterogeneity_ = 0.001), respectively (Figure S2).

The associations of menopausal HRT with the risk of ovarian cancer in various histological subtypes are summarized in Table 3.

**Table 3:** Summary risk estimates of the association between hormone replacement therapy and ovarian cancer by histologic subtype.

**Histologic subtype of ovarian cancer**	**Subgroup**	**No. of studies**	**Pooled RR(95% CI)**	***I*^**2**^ (%)**	***P*_***heterogeneity***_**
Serous tumor	All studies	12	1.50 (1.35–1.68)	27.5	0.175
	Study design				
	Cohort studies	3	1.64 (1.46–1.85)	0.0	0.998
	Case control studies	9	1.42 (1.20–1.67)	33.6	0.149
	Continent				
	North America	6	1.61 (1.38–1.88)	0.0	0.578
	Europe	5	1.51 (1.36–1.68)	2.7	0.391
	Australia	1	0.71 (0.40–1.27)	NA	NA
	Hormones types				
	HRT	9	1.48 (1.29–1.70)	38.8	0.109
	ERT	3	1.54 (1.25–1.89)	4.3	0.352
Mucinous	All studies	9	0.66 (0.52–0.85)	0.0	0.553
tumor	Study design			
	Cohort studies	2	0.71 (0.50–1.01)	0.0	0.495
	Case control studies	7	0.62 (0.44–0.89)	1.9	0.410
	Continent				
	North America	4	0.79 (0.43–1.44)	0.0	0.666
	Europe	4	0.62 (0.40–0.94)	38.8	0.179
	Australia	1	0.67 (0.28–1.62)	NA	NA
	Hormones types				
	HRT	6	0.74 (0.58–0.97)	0.0	0.900
	ERT	3	0.41 (0.23–0.73)	0.0	0.383
Endometrioid	All studies	10	1.48 (1.13–1.94)	51.8	0.028
tumor	Study design				
	Cohort studies	3	1.88 (1.49–2.36)	0.0	0.789
	Case control studies	7	1.25 (0.86–1.82)	53.1	0.046
	Continent				
	North America	6	1.32 (0.78–2.23)	66.2	0.011
	Europe	4	1.61 (1.32–1.97)	5.5	0.365
	Hormones types				
	HRT	7	1.40 (0.99–1.98)	60.2	0.020
	ERT	3	1.68 (0.97–2.91)	38.6	0.196
Clear cell	All studies	5	0.94 (0.65–1.36)	0.0	0.689
tumor	Study design				
	Cohort studies	2	1.06 (0.50–2.23)	47.3	0.168
	Case control studies	3	0.95 (0.48–1.90)	0.0	0.837
	Continent				
	North America	3	1.36 (0.70–2.64)	0.0	0.776
	Europe	2	0.80 (0.51–1.24)	0.0	0.853
	Hormones types				
	HRT	4	0.97 (0.66–1.44)	0.0	0.567
	ERT	1	0.72 (0.23–2.27)	NA	NA
Other type of	All studies	5	1.21 (0.91–1.61)	39.0	0.161
ovarian cancer	Study design				
	Cohort studies	1	1.53 (1.10–2.12)	NA	NA
	Case control studies	4	1.08 (0.80–1.45)	16.2	0.311
	Continent				
	North America	3	1.44 (1.09–1.92)	0.0	0.747
	Europe	2	1.07 (0.62–1.84)	69.6	0.070
	Hormones types				
	HRT	4	1.44 (1.13–1.84)	0.0	0.900
	ERT	1	0.82 (0.56–1.20)	NA	NA

*RR, relative risk; CI, confidence interval*.

In the five major histologic subtypes, significant positive associations were observed in serous (RR = 1.50, 95%CI 1.35–1.68, *I*^2^ = 27.5%, *P*_heterogeneity_ = 0.175) and endometrioid (RR = 1.48, 95%CI 1.13–1.94, *I*^2^ = 51.8%, *P*_heterogeneity_ = 0.028) tumors. In subgroup analysis stratified by study design, pooled RRs for serous tumors in cohort and case-control studies were 1.64 (95%CI 1.46–1.85, *I*^2^ = 0.0%, *P*_heterogeneity_ = 0.998) and 1.42 (95%CI 1.20–1.67, *I*^2^ = 33.6%, *P*_heterogeneity_ = 0.149), respectively. In subgroup analysis stratified by continent, significant positive associations were found for North America (RR = 1.61, 95%CI 1.38–1.88, *I*^2^ = 0.0%, *P*_heterogeneity_ = 0.578) and Europe (RR = 1.51, 95%CI 1.36–1.68, *I*^2^ = 2.7%, *P*_heterogeneity_ = 0.391), respectively. In subgroup analysis stratified by the hormones types, pooled RRs for HRT and ERT were 1.48 (95%CI 1.29–1.70, *I*^2^ = 38.8%, *P*_heterogeneity_ = 0.109) and 1.54 (95%CI 1.25–1.89, *I*^2^ = 4.3%, *P*_heterogeneity_ = 0.352), respectively. In subgroup analysis for endometrioid tumors, significant positive associations were obtained in cohort studies (RR = 1.88, 95%CI 1.49–2.36, *I*^2^ = 0.0%, *P*_heterogeneity_ = 0.789) and Europe (RR = 1.61, 95%CI 1.32–1.97, *I*^2^ = 5.5%, *P*_heterogeneity_ = 0.365), respectively.

### Meta-Regression and Sensitivity Analysis

To assess between-study heterogeneity, we performed univariate meta-regression with the covariates of study design, publication year and continent. However, none of these covariates was found to have a significant impact on between-study heterogeneity. After excluding two study (16, 20) (RR > 3.0) in the ovarian cancer assessment, the heterogeneity remained at a moderate level (*I*^2^ = 52.1%, *P*_heterogeneity_ < 0.001), and the pooled RR was 1.27 (95%CI 1.17–1.37).

In sensitivity analysis excluding one study at a time, pooled RRs (95%CIs) of the association of menopausal HRT with the risk of ovarian cancer ranged from 1.27 (95%CI 1.18–1.38) to 1.31 (95%CI 1.20–1.42). No individual study had excessive effect on the pooled RR.

### Publication Bias

Visual inspection of the funnel plot (Figure S3) and Egger's test (*P*_ovarian cancer_ = 0.083) showed no evidence of significant small-study effect for the association of menopausal HRT with the risk of ovarian cancer. Egger's test also provided no evidence of significant small-study effect for the association of menopausal HRT with the risk of ovarian cancer by histologic subtype (*P*_serous tumor_ = 0.762, *P*_endometrioid tumor_ = 0.550, *P*_mucinous tumor_ = 0.655, *P*_clear call tumor_ = 0.349, *P*_other types of ovarian cancer_ = 0.892).

## Discussion

The current meta-analysis assessed associations of menopausal HRT with the risk of ovarian cancer in various histologic subtypes. Findings of this meta-analysis indicated a positive association of menopausal HRT with the risk of ovarian cancer. In subgroup analysis by study design, significant positive associations were observed in both cohort and case control studies. In subgroup analysis by histologic subtypes, we found that menopausal HRT may increase the risk of serous and endometrioid tumors. In subgroup analysis by the hormones types, significant positive associations were observed for both HRT and ERT. The pooled RR indicated that there might be a stronger association in ERT users, but the result might be not true enough with the insufficient studies about ERT.

The mechanism underlying the association of menopausal HRT with ovarian cancer is not well-understood. A theory suggests that high levels of gonadotropins during menopause act as a promoter on the affected ovarian tissue (49). These findings imply that menopausal HRT might decrease the risk of cancer by reducing the levels of gonadotropins. However, these benefits might be outweighed by estrogen-induced ovarian cell proliferation (50). Estrogen and progesterone receptors are found in normal ovarian surface and most of ovarian tumors are estrogen receptor-positive (51, 52). Estrogen could stimulate the proliferation of ovarian surface epithelial cells and progesterone could promote the apoptosis of ovarian cells. The weaker risk effect of HRT than ERT may be because progesterone counteract the proliferative effect of estrogen on ovarian cells (52–54).

Between-study heterogeneity is common in meta-analysis. It is necessary to explore the potential sources of between-study heterogeneity. In this meta-analysis, a moderate between-study heterogeneity was found. However, meta-regression analysis with the covariates of study design, published year and continent revealed no source of between-study heterogeneity. After excluding two study (20) (RR > 3.0) in the analysis of menopausal HRT and ovarian cancer, between-study heterogeneity was slightly reduced, but results did not change substantially. This indicated that the results were stable and credible.

This meta-analysis had some advantages. The first is the sufficient sample size that made the study had high statistical power to detect even small associations. Secondly, we extracted RRs reflecting the highest degree of control for potential confounders in the original studies. This will help us to get a real connection between the factors and the disease. Thirdly, sensitivity analysis showed that no individual study had excessive effects on pooled data for menopausal HRT and the risk of ovarian cancer by histologic subtypes. Fourthly, after excluding two study (RR > 3.0) in ovarian cancer analysis, between-study heterogeneity was slightly reduced, and the results did not change substantially, suggesting that they were stable. Fifthly, in subgroup analysis stratified by the hormones types, we found both HRT and ERT could increase the risk of ovarian cancer.

However, there were still some deficiencies in this meta-analysis. First, the authors adjusted for confounders such as age, parity, duration of oral contraceptive use, tubal ligation, age at natural menopause and age at menarche etc. in original studies, but we dare not deny whether some unknown confounders might lead to exaggerating or underestimating the association. In addition, confounders adjusted for in various studies were different, which might affect the observed association. Some common biases such as selection bias, recall bias and lost to follow-up etc. in observational studies might also affect the authenticity of the results. Secondly, follow-up durations in various cohort studies differed. Some potential cases might not be observed due to limited follow-up in certain studies. Thirdly, menopausal HRT might be slightly different in each of the papers analyzed. In some papers, the HRT referred to estorgens + progestins, but in others, the HRT referred to only estorgens or estorgens + progestins. This might affect the observed association. Fourthly, the limited amount of studies assessing histologic subtypes made it difficult to confirm the relationship in terms of the kind of therapy and its association with the histological subtypes of ovarian cancer. Fifthly, although the age of subjects was over 50 years old in most included studies, a few studies covered the data from women from pre-menopausal age. This might bias the results of the meta-analysis. Sixthly, the insufficient available data prevented us from conducting a dose-response relationship to explore the association between length of HRT use and the risk of ovarian cancer.

In conclusion, this meta-analysis suggests that menopausal HRT may increase the risk of ovarian cancer, especially for serous and endometrioid tumors. This finding requires confirmation by further studies of associations of menopausal HRT with the risk of ovarian cancer in various histological subtypes.

## Data Availability Statement

All datasets for this study are included in the article/supplementary material.

## Author Contributions

YL and LM conceived and coordinated the study, designed, performed and analyzed the experiments, and wrote the paper. XY, JB, DL, CS, and JZ carried out the data collection, data analysis, and revised the paper. YM and JL designed the study, carried out the data analysis, and revised the paper. All authors reviewed the results and approved the final version of the manuscript.

### Conflict of Interest

The authors declare that the research was conducted in the absence of any commercial or financial relationships that could be construed as a potential conflict of interest.

## Footnotes

^1^https://gco.iarc.fr/today/data/factsheets/cancers/25-Ovary-fact-sheet.pdf

## Supplementary Material

Figure S1 Forest plot of menopausal HRT and the risk of ovarian cancer in subgroup analysis stratified by geographic location. The size of a gray box is proportional to the weight assigned to the respective study, and horizontal lines represent 95% confidence intervals (CIs).

Figure S2 Forest plot of menopausal HRT and the risk of ovarian cancer in subgroup analysis stratified by the hormones types. The size of a gray box is proportional to the weight assigned to the respective study, and horizontal lines represent 95% confidence intervals (CIs).

Figure S3 The funnel plot of menopausal HRT and the risk of ovarian cancer. Each dot represents a distinct study.

## References

[1] Siegel R Miller K Jemal A Cancer statistics, 2018. CA Cancer J Clin. (2018) 68:7–30. 10.3322/caac.21442 29313949

[2] Li DP Du C Zhang ZM Li GX Yu ZF Wang X. Breastfeeding and ovarian cancer risk: a systematic review and meta-analysis of 40 epidemiological studies. Asian Pac J Cancer Prev. (2014) 15:4829–37. 10.7314/APJCP.2014.15.12.4829 24998548

[3] Havrilesky LJ Moorman PG Lowery WJ Gierisch JM Coeytaux RR Urrutia RP. Oral contraceptive pills as primary prevention for ovarian cancer: a systematic review and meta-analysis. Obstet Gynecol. (2013) 122:139–47. 10.1097/AOG.0b013e318291c235 23743450

[4] Bae HS Kim HJ Hong JH Lee JK Lee NW Song JY. Obesity and epithelial ovarian cancer survival: a systematic review and meta-analysis. J Ovarian Res. (2014) 7:41. 10.1186/1757-2215-7-41 24834130 PMC4022349

[5] Liu Z Zhang TT Zhao JJ Qi SF Du P Liu DW. The association between overweight, obesity and ovarian cancer: a meta-analysis. Jpn J Clin Oncol. (2015) 45:1107–15. 10.1093/jjco/hyv150 26491203

[6] Lee JY Jeon I Kim JW Song YS Yoon JM Park SM. Diabetes mellitus and ovarian cancer risk: a systematic review and meta-analysis of observational studies. Int J Gynecol Cancer. (2013) 23:402–12. 10.1097/IGC.0b013e31828189b2 23354371

[7] Braem MG Onland-Moret NC Schouten LJ Kruitwagen RF Lukanova A Allen NE. Multiple miscarriages are associated with the risk of ovarian cancer: results from the European Prospective Investigation into Cancer and Nutrition. PLoS ONE. (2012) 7:e37141. 10.1371/journal.pone.0037141 22623987 PMC3356371

[8] Kazerouni N Greene MH Lacey JV Jr Mink PJ Schairer C. Family history of breast cancer as a risk factor for ovarian cancer in a prospective study. Cancer. (2006) 107:1075–83. 10.1002/cncr.22082 16881078

[9] Wang K Li F Chen L Lai YM Zhang X Li HY. Change in risk of breast cancer after receiving hormone replacement therapy by considering effect-modifiers: a systematic review and dose-response meta-analysis of prospective studies. Oncotarget. (2017) 8:81109–24. 10.18632/oncotarget.20154 29113371 PMC5655266

[10] Sillero-Arenas M Delgado-Rodriguez M Rodigues-Canteras R Bueno-Cavanillas A Galvez-Vargas R. Menopausal hormone replacement therapy and breast cancer: a meta-analysis. Obstet Gynecol. (1992) 79:286–94. 1530988

[11] Folsom A Anderson J Ross J. Estrogen replacement therapy and ovarian cancer. Epidemiology. (2004) 15:100–4. 10.1097/01.ede.0000091606.31903.8e 14712153

[12] Perri T Lifshitz D Sadetzki S Oberman B Meirow D Ben-Baruch G Fertility treatments and invasive epithelial ovarian cancer risk in Jewish Israeli BRCA1 or BRCA2 mutation carriers. Fertil Steril. (2015) 103:1305–12. 10.1016/j.fertnstert.2015.02.011 25792249

[13] Morch L Lokkegaard E Andreasen A Kruger-Kjaer S Lidegaard O. Hormone therapy and ovarian cancer. JAMA. (2009) 302:298–305. 10.1001/jama.2009.1052 19602689

[14] Urban N Hawley S Janes H Karlan BY Berg CD Drescher CW. Identifying post-menopausal women at elevated risk for epithelial ovarian cancer. Gynecol Oncol. (2015) 139:253–60. 10.1016/j.ygyno.2015.08.024 26343159 PMC4664187

[15] Tavani A Ricci E La Vecchia C Surace M Benzi G Parazzini F Influence of menstrual and reproductive factors on ovarian cancer risk in women with and without family history of breast or ovarian cancer. Int J Epidemiol. (2000) 29:799–802. 10.1093/ije/29.5.799 11034959

[16] Lacey JV Jr Mink PJ Lubin JH Sherman ME Troisi R Hartge P. Menopausal hormone replacement therapy and risk of ovarian cancer. JAMA. (2002) 288:334–41. 10.1001/jama.288.3.334 12117398

[17] Simin J Tamimi R Lagergren J Adami HO Brusselaers N. Menopausal hormone therapy and cancer risk: an overestimated risk? Eur J Cancer. (2017) 84:60–8. 10.1016/j.ejca.2017.07.012 28783542

[18] Rossing MA Cushing-Haugen KL Wicklund KG Doherty JA Weiss NS. Menopausal hormone therapy and risk of epithelial ovarian cancer. Cancer Epidemiol Biomarkers Prev. (2007) 16:2548–56. 10.1158/1055-9965.EPI-07-0550 18086757

[19] Beral V Million Women Study C Bull D Green J Reeves G. Ovarian cancer and hormone replacement therapy in the Million Women Study. Lancet. (2007) 369:1703–10. 10.1016/S0140-6736(07)60534-0 17512855

[20] Polychronopoulou A Tzonou A Hsieh CC Kaprinis G Rebelakos A Toupadaki N. Reproductive variables, tobacco, ethanol, coffee and somatometry as risk factors for ovarian cancer. Int J Cancer. (1993) 55:402–7. 10.1002/ijc.2910550312 8375923

[21] Hempling RE Wong C Piver MS Natarajan N Mettlin CJ. Hormone replacement therapy as a risk factor for epithelial ovarian cancer: results of a case-control study. Obstet Gynecol. (1997) 89:1012–6. 10.1016/S0029-7844(97)00118-X 9170483

[22] Kotsopoulos J Lubinski J Neuhausen SL Lynch HT Rosen B Ainsworth P. Hormone replacement therapy and the risk of ovarian cancer in BRCA1 and BRCA2 mutation carriers. Gynecol Oncol. (2006) 100:83–8. 10.1016/j.ygyno.2005.07.110 16137751

[23] Purdie DM Bain CJ Siskind V Russell P Hacker NF Ward BG. Hormone replacement therapy and risk of epithelial ovarian cancer. Br J Cancer. (1999) 81:559–63. 10.1038/sj.bjc.6690731 10507786 PMC2362907

[24] Sit AS Modugno F Weissfeld JL Berga SL Ness RB. Hormone replacement therapy formulations and risk of epithelial ovarian carcinoma. Gynecol Oncol. (2002) 86:118–23. 10.1006/gyno.2002.6746 12144815

[25] Li K Husing A Fortner RT Tjonneland A Hansen L Dossus L. An epidemiologic risk prediction model for ovarian cancer in Europe: the EPIC study. Br J Cancer. (2015) 112:1257–65. 10.1038/bjc.2015.22 25742479 PMC4385951

[26] Bakken K Alsaker E Eggen AE Lund E. Hormone replacement therapy and incidence of hormone-dependent cancers in the Norwegian Women and Cancer study. Int J Cancer. (2004) 112:130–34. 10.1002/ijc.20389 15305384

[27] Wernli KJ Newcomb PA Hampton JM Trentham-Dietz A Egan KM. Hormone therapy and ovarian cancer: incidence and survival. Cancer Causes Control. (2008) 19:605–13. 10.1007/s10552-008-9125-x 18264784 PMC2729759

[28] Koskela-Niska V Lyytinen H Riska A Pukkala E Ylikorkala O. Ovarian cancer risk in postmenopausal women using estradiol-progestin therapy - a nationwide study. Climacteric. (2013) 16:48–53. 10.3109/13697137.2012.663818 22640598

[29] Rasmussen ELK Hannibal CG Dehlendorff C Baandrup L Junge J Vang R. Parity, infertility, oral contraceptives, and hormone replacement therapy and the risk of ovarian serous borderline tumors: a nationwide case-control study. Gynecol Oncol. (2017) 144:571–6. 10.1016/j.ygyno.2017.01.002 28108026

[30] Bethea TN Palmer JR Adams-Campbell LL Rosenberg L. A prospective study of reproductive factors and exogenous hormone use in relation to ovarian cancer risk among Black women. Cancer Causes Control. (2017) 28:385–91. 10.1007/s10552-016-0840-4 28025764 PMC5386825

[31] Chiaffarino F Pelucchi C Parazzini F Negri E Franceschi S. Reproductive and hormonal factors and ovarian cancer. Ann Oncol. (2001) 12:337–41. 10.1023/A:1011128408146 11332145

[32] Braem MG Onland-Moret NC van den Brandt PA Goldbohm RA Peeters PH Kruitwagen RF. Reproductive and hormonal factors in association with ovarian cancer in the Netherlands cohort study. Am J Epidemiol. (2010) 172:1181–9. 10.1093/aje/kwq264 20861144 PMC2970782

[33] Pasalich M Su D Binns CW Lee AH. Reproductive factors for ovarian cancer in southern Chinese women. J Gynecol Oncol. (2013) 24:135–40. 10.3802/jgo.2013.24.2.135 23653830 PMC3644689

[34] Salazar-Martinez E Lazcano-Ponce EC Gonzalez Lira-Lira G Escudero-De los Rios P Salmeron-Castro J Hernandez-Avila M. Reproductive factors of ovarian and endometrial cancer risk in a high fertility population in Mexico. Cancer Res. (1999) 59:3658–62. 10446978

[35] Jordan SJ Green AC Whiteman DC Webb PM. Risk factors for benign serous and mucinous epithelial ovarian tumors. Obstet Gynecol. (2007) 109:647–54. 10.1097/01.AOG.0000254159.75977.fa 17329516

[36] Adami HO Persson I Hoover R Schairer C Bergkvist L. Risk of cancer in women receiving hormone replacement therapy. Int J Cancer. (1989) 44:833–9. 10.1002/ijc.2910440515 2583865

[37] Schneider C Jick SS Meier CR Risk of gynecological cancers in users of estradiol/dydrogesterone or other HRT preparations. Climacteric. (2009) 12:514–24. 10.3109/13697130903075352 19905903

[38] Soegaard M Jensen A Hogdall E Christensen L Hogdall C Blaakaer J. Different risk factor profiles for mucinous and nonmucinous ovarian cancer: results from the Danish MALOVA study. Cancer Epidemiol Biomarkers Prev. (2007) 16:1160–6. 10.1158/1055-9965.EPI-07-0089 17548679

[39] Koskela-Niska V Pukkala E Lyytinen H Ylikorkala O Dyba T. Effect of various forms of postmenopausal hormone therapy on the risk of ovarian cancer–a population-based case control study from Finland. Int J Cancer. (2013) 133:1680–8. 10.1002/ijc.28167 23526244

[40] Risch HA. Estrogen replacement therapy and risk of epithelial ovarian cancer. Gynecol Oncol. (1996) 63:254–7. 10.1006/gyno.1996.0315 8910636

[41] Mills PK Riordan DG Cress RD Goldsmith DF. Hormone replacement therapy and invasive and borderline epithelial ovarian cancer risk. Cancer Detect Prev. (2005) 29:124–32. 10.1016/j.cdp.2004.11.002 15829372

[42] Riman T Dickman PW Nilsson S Correia N Nordlinder H Magnusson CM. Hormone replacement therapy and the risk of invasive epithelial ovarian cancer in Swedish women. J Natl Cancer Inst. (2002) 94:497–504. 10.1093/jnci/94.7.497 11929950

[43] Morch LS Lokkegaard E Andreasen AH Kjaer SK Lidegaard O. Hormone therapy and different ovarian cancers: a national cohort study. Am J Epidemiol. (2012) 175:1234–42. 10.1093/aje/kwr446 22517811

[44] Moorman PG Schildkraut JM Calingaert B Halabi S Berchuck A. Menopausal hormones and risk of ovarian cancer. Am J Obstet Gynecol. (2005) 193:76–82. 10.1016/j.ajog.2004.11.013 16021062

[45] Yang HP Trabert B Murphy MA Sherman ME Sampson JN Brinton LA. Ovarian cancer risk factors by histologic subtypes in the NIH-AARP Diet and Health Study. Int J Cancer. (2012) 131:938–48. 10.1002/ijc.26469 21960414 PMC3505848

[46] Danforth KN Tworoger SS Hecht JL Rosner BA Colditz GA Hankinson SE. A prospective study of postmenopausal hormone use and ovarian cancer risk. Br J Cancer. (2007) 96:151–6. 10.1038/sj.bjc.6603527 17179984 PMC2360221

[47] Greiser CM Greiser EM Doren M. Menopausal hormone therapy and risk of ovarian cancer: systematic review and meta-analysis. Hum Reprod Update. (2007) 13:453–63. 10.1093/humupd/dmm012 17573406

[48] Pearce CL Chung K Pike MC Wu AH. Increased ovarian cancer risk associated with menopausal estrogen therapy is reduced by adding a progestin. Cancer. (2009) 115:531–9. 10.1002/cncr.23956 19127543 PMC4203480

[49] Stadel BV. Letter: The etiology and prevention of ovarian cancer. Am J Obstet Gynecol. (1975) 123:772–4. 10.1016/0002-9378(75)90509-8 1200073

[50] Cramer DW Welch WR. Determinants of ovarian cancer risk. II. Inferences regarding pathogenesis. J Natl Cancer Inst. (1983) 71:717–21. 6578367

[51] Cunat S Hoffmann P Pujol P. Estrogens and epithelial ovarian cancer. Gynecol Oncol. (2004) 94:25–32. 10.1016/j.ygyno.2004.03.026 15262115

[52] Syed V Ulinski G Mok SC Yiu GK Ho SM. Expression of gonadotropin receptor and growth responses to key reproductive hormones in normal and malignant human ovarian surface epithelial cells. Cancer Res. (2001) 61:6768–76. 11559549

[53] Lau KM Mok SC Ho SM. Expression of human estrogen receptor-alpha and -beta, progesterone receptor, and androgen receptor mRNA in normal and malignant ovarian epithelial cells. Proc Natl Acad Sci USA. (1999) 96:5722–7. 10.1073/pnas.96.10.5722 10318951 PMC21927

[54] Mukherjee K Syed V Ho SM. Estrogen-induced loss of progesterone receptor expression in normal and malignant ovarian surface epithelial cells. Oncogene. (2005) 24:4388–400. 10.1038/sj.onc.1208623 15806153

