# Supplementary figures and images for: Menopausal Hormone Replacement Therapy and the Risk of Ovarian Cancer: A Meta-Analysis
Source: Front Endocrinol (Lausanne). 2019 Dec 3;10:801. doi: 10.3389/fendo.2019.00801 (PMC6902084; doi:10.3389/fendo.2019.00801)

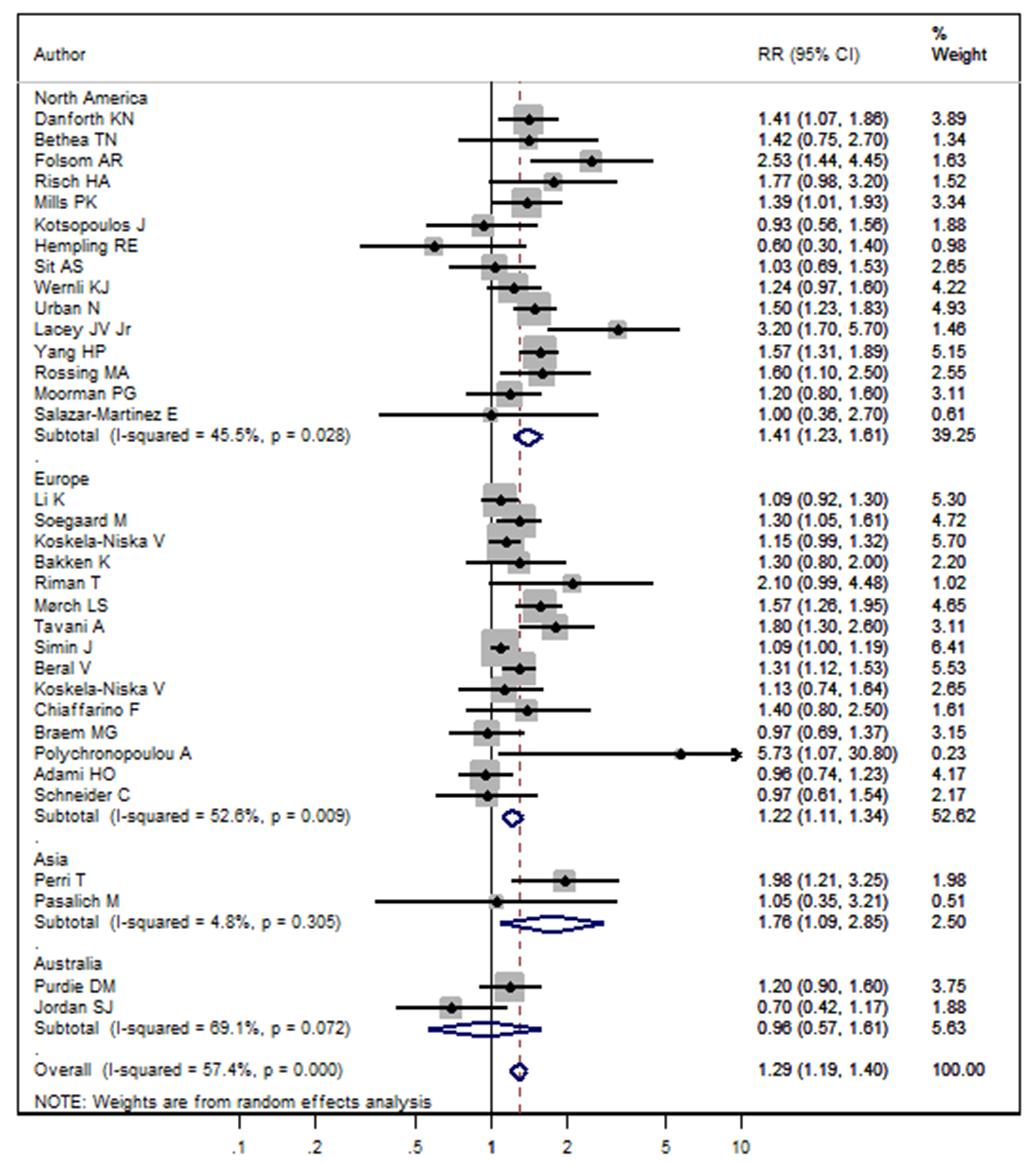

Supplement: Figure S1 — Forest plot of menopausal HRT and the risk of ovarian cancer in subgroup analysis stratified by geographic location. The size of a gray box is proportional to the weight assigned to the respective study, and horizontal lines represent 95% confidence intervals (CIs). [file Image_1.TIF]

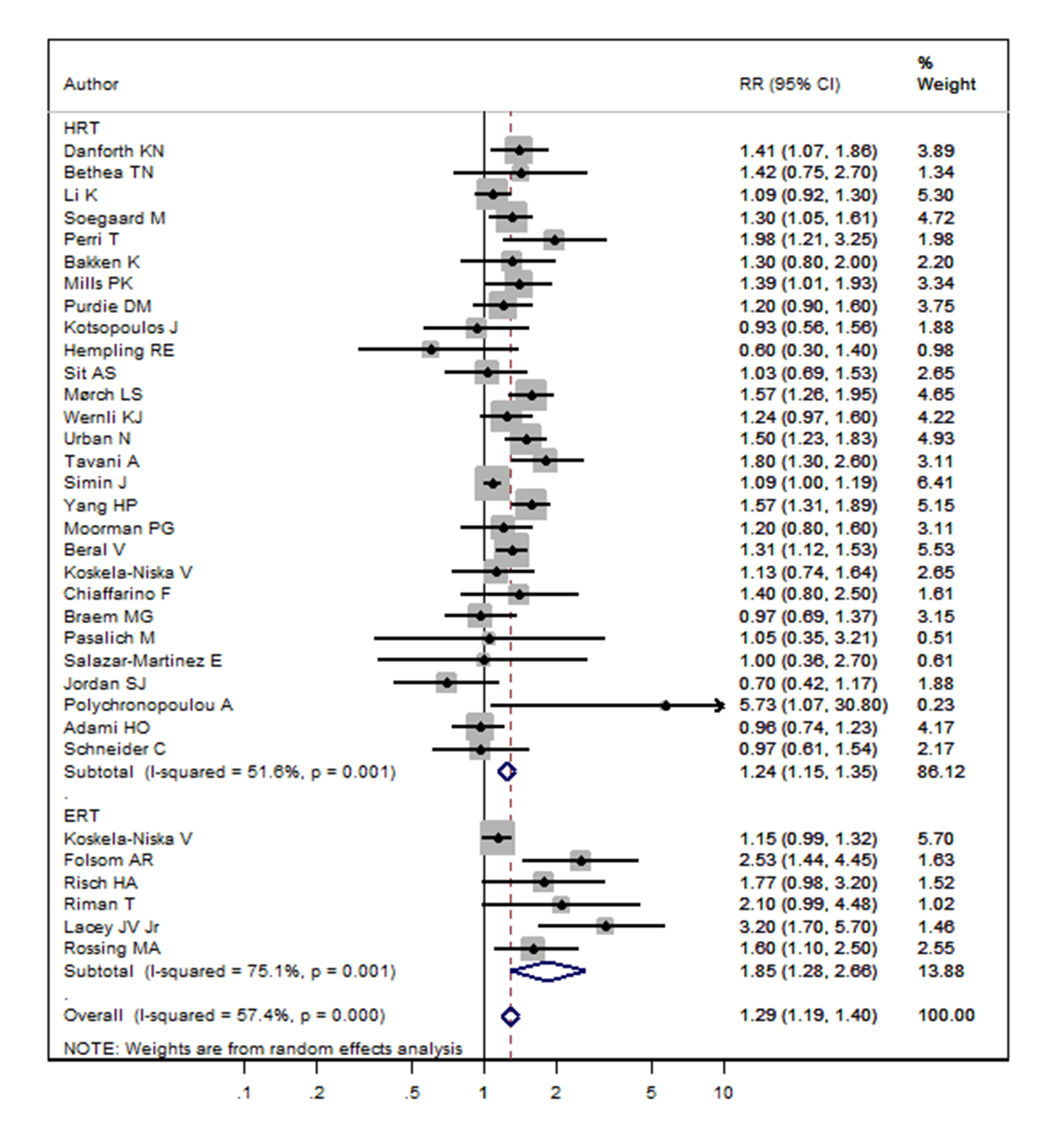

Supplement: Figure S2 — Forest plot of menopausal HRT and the risk of ovarian cancer in subgroup analysis stratified by the hormones types. The size of a gray box is proportional to the weight assigned to the respective study, and horizontal lines represent 95% confidence intervals (CIs). [file Image_2.TIF]

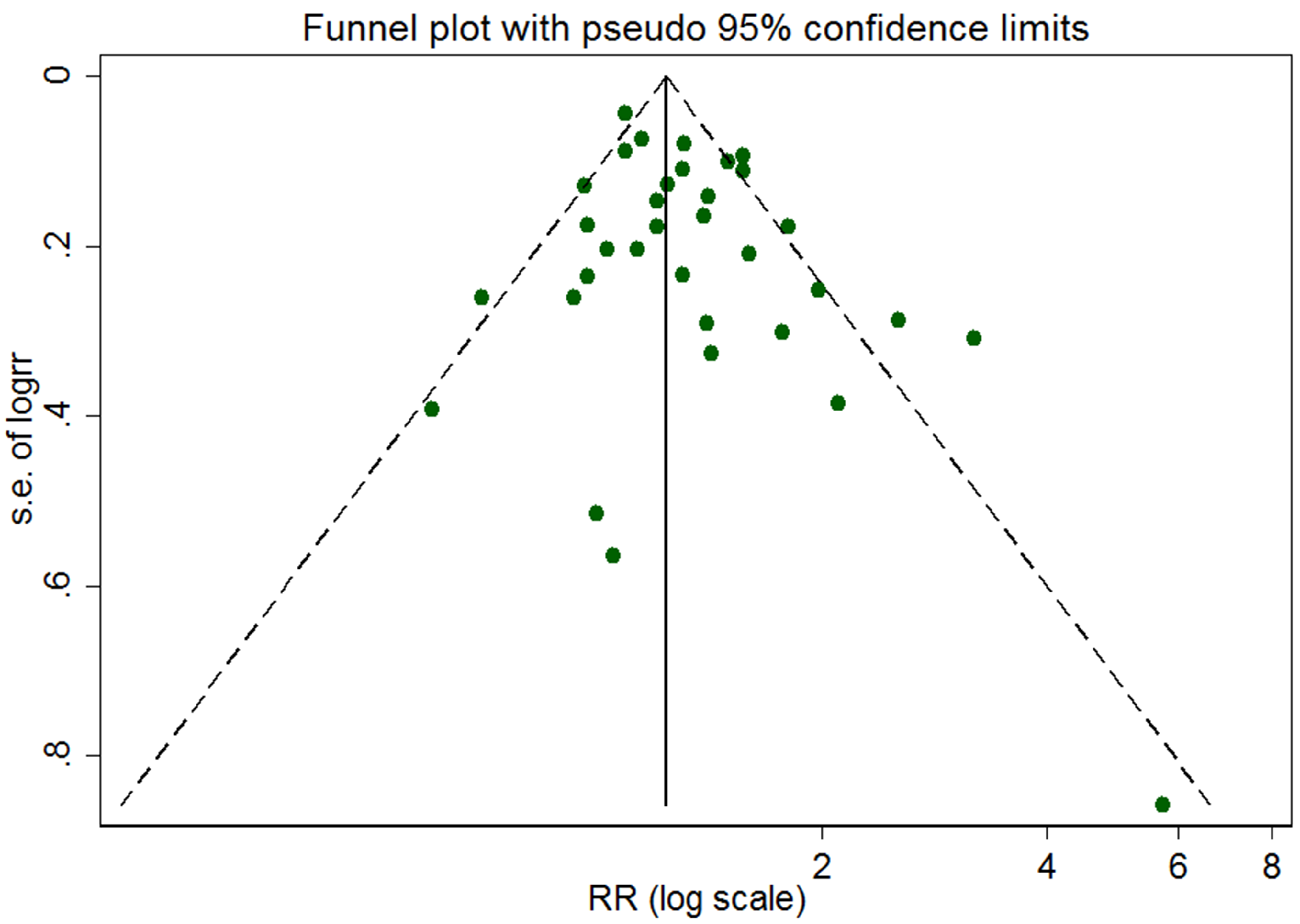

Supplement: Figure S3 — The funnel plot of menopausal HRT and the risk of ovarian cancer. Each dot represents a distinct study. [file Image_3.TIF]
